# Notes on Pernicious Malarial Fever*Read to the Madison County Medical Society, and published by request of the Society.Resolved by Madison County Medical Society, That the paper read by Dr. J. D. Jordon before said society, subject: “Pernicious Malaria,” be sent to Dr. Daniel for publication in the Texas Medical Journal.(Signed)Benj. F. Gibson, M. D., President.Orlando Patton, M. D., Secretary.

**Published:** 1904-11

**Authors:** J. D. Jordan

**Affiliations:** Madisonville, Texas


### TEXAS MEDICAL JOURNAL.

ESTABLISHED JULY, 1885.

PUBLISHED MONTHLY.—SUBSCRIPTION $1.00 A YEAR.

VOL. XX. AUSTIN, NOVEMBER, 1904. No. 5.

For Texas Medical Journal.

### Notes on Pernicious Malarial Fever.*

BY J. D. JORDAN, M. D., MADISONVILLE, TEXAS.

At the request of the program committee, I submit for your consideration the following rambling comments upon the subject selected by you for discussion.

The theme is timely. The prevalence of these diseases is usually expected after a hot, rainy season. Heat and moisture are condi-
It is not explained why the *plasmodium malariæ* does not seek entrance into polite society through other varieties of mosquitoes; door unfastened; whatever the means resorted to, the object is booty; so, likewise, the malarial burglar may enter the human house though the kind offices of his friend, anopheles, in search of booty. The booty in the last instance is blood corpuscles.
tions essential to the production of the *plasmodium malariæ* and its concomitant host, the *anopheles claviger*. Burglars enter unprotected houses through various devices: Sometimes, by violent means, when the inhabitants are out of town; sometimes, through the carelessness or connivance of servants in leaving a window or a or, through their kinsman, the gnat; or, the festive flea; or, the tick; or, the chique; or, that two familiar companion of the night that drives away "nature's sweet restorer"—and, although he "has no wings, gets there all the same." It would be well to place under the ban the whole generation of pestiferous insects. Let them be *anathema—maranatha!*

---

*Read to the Madison County Medical Society, and published by request of the Society.

Resolved by Madison County Medical Society, That the paper read by Dr. J. D. Jordon before said society, subject: "Pernicious Malaria," be sent to Dr. Daniel for publication in the *Texas Medical Journal.*

(Signed) BENJ. F. GIBSON, M. D., President.
ORLANDO PATTON, M. D., Secretary.

The burglarious *hemasporidia,* when housed safely, may begin operations at once; or, may wait his time in patience for days, weeks or months for a suitable occasion; only now and then nibbling a stray corpuscle. When the premises please and the opportunity offers, he will begin to increase the family at such a rapid rate as to drive to the winds all hope of extinction by race suicide. New colonies are sent out in all directions with marvelous rapidity. The spleen, the liver, the brain, the blood, the kidneys—in fact, every available portion of the anatomy, even the marrow of the bones, is invaded. The whole blood-manufacturing organs of the body working over-time, night and day, can hardly manufacture blood corpuscles enough to satisfy their voracious appetites.

The two-legged burglar may, from habit, observe the ordinary rules of decency; not so with his malarial prototype. The latter is most likely to deposit his excrement without the least regard for your ideas of cleanliness. This excreta, under the name of toxins, is supposed to cause that discharge of nervous energy called the malarial paroxysm.

Let us now purloin and condense a few ideas from the *Encyclopedia of Practical Medicine:*

"The infecting agent causing malarial fever is a parasite belonging to the class sporozoa of the order of hemosporidia. They may be divided into three classes: First. The tertian. Second. The quartan. Third. The estivo-autumnal. The first two are associated with more or less regularly intermittent fever; the last variety may be regularly intermittent, but is more often irregular or continued.

"The quartan parasite exists in the blood in great groups, all the members of which are approximately at the same stage of development and pass through their cycle nearly in unison. The cycle lasts almost exactly seventy-two hours, at the end of which time the parasites which have reached maturity undergo sporulation; the fresh sporules attacking new corpuscles. Sporulation of a group of parasites, if it has reached sufficient size, is nearly always associated with the paroxysm of the individual. The immediate cause of the malarial paroxysm is thought to be the liberation of some toxic substances by the parasites.

"Not infrequently, two or three groups of parasites are present, when they almost always reach maturity on different days, resulting in paroxysms on two different days, with a day of intermission between; or, in daily paroxysms. Very early the presence of multiple groups causes irregular or continued fever.

"Second. The tertian parasite also exists in the blood in great groups. Its life cycle lasts forty-eight hours, so that sporulation occurs every other day. Infection with two groups is common, giving rise to every day paroxysms. Infection with multiple groups giving rise to irregular or continued fever occurs but rarely.

"Third. The third form of the malarial parasite—estivo-autumnal—differs from the other varieties in not being arranged in groups, except in the beginning of an attack. This is the group that usually causes continued, remittent fever, which is not uncommonly mistaken for typhoid.

"In all these forms of parasites, delicate, motile filaments develop from certain full-grown forms. These filaments are supposed to be sexual elements, or the elements of reproduction. Penetration of the cell of the sporozoa by the filament being the method of sexual congress. It is only the fecundated bodies that are capable of undergoing further development in the body of the mosquito."

Minute description of all the varieties of plasmodium malariæ may be found in any recent work on medical microscopy. Each variety, when developed in sufficient numbers, produces a set of symptoms peculiar to itself—which you will find more fully described in works on practice—as well as the pathological effects of their ravages. If it is true that the malarial parasite is introduced into the circulation through the mosquito only—and science has demonstrated no other way—it is obvious that protective measures consist in prohibiting personal contact with the insect. How best to destroy these pests has already been explained by your very capable State Health Officer.

The injurious effects of the parasite on the human system may depend upon its condition, its powers of resistance and the number of parasites at work. For the present, our subject limits us to the pernicious variety of the disease which may be produced by all forms of the parasite.

Pernicious malarial remittent may begin with a chill of short duration, to be followed by high temperature for twenty-four, forty-eight or seventy-two hours, when a decided remission—not *intermission*—of fever from three to twelve hours, or longer, occurs. During the pyrexia, the skin is hot; the face flushed; the pulse strong; severe pains are felt in muscles of the back and extremities; and headaches are present. Great nervous irritability is shown by restlessness, vomiting and delirium. Constipation usually exists. The temperature of the body may range from 101° to 110°—very high temperature indicates impending fatal issue. At the close of the period of pyrexia, a remission occurs; during which the temperature falls to the neighborhood of 100°; the nervous irritability indicated by the headache, muscular pains, delirium, subsides; the skin becomes moist and sometimes the perspiration is very free. The patient becomes calm, conscious and almost comfortable. During this time, persons not familiar with the disease are induced to believe that the patient is on the high road to recovery. In a short time, however—say, from three to twelve hours—maybe a little longer—this delusion is dispelled by a return of all the distressing symptoms, the initial chill excepted. The second exascerbation will continue for about the same length of time as the first, viz., about twenty-four, forty-eight or seventy-two hours. Each recurring remission is apt to be less pronounced and of shorter duration than the former one, until it becomes hardly perceptible. The disease may continue in mild cases for from three days to three weeks; or even longer in severe cases.

It is erroneous to assume that every case of fever that does not yield to quinine in a week is typhoid. Pernicious, remittent fever may continue for weeks. I would not like to prescribe the exact limit. If a re-introduction of parasites is allowed and the only reliable remedy for their extinction withheld, why should the disease continue until tired nature ceases to struggle against the inevitable? But to go on with the description. The skin and whites of the eyes often show a jaundiced hue. The tongue becomes coated with a brown fur. In protracted cases a set of symptoms may ensue, usually characterized as the typhoid state—marked by a temperature of 100° to 102°, restlessness, vigilance, muttering delirium, picking at the bedclothes, coma, vigal, dry, cracked tongue, sordes indicating great prostration of vital powers. The bowels may be loose or constipated. Hemorrhages may occur from the bowels, urinary or respiratory organs. Pernicious malarial fever may be distinctly intermittent—either quotidian or tertian. The algid form is characterized by pallor and coldness of the external surface; the countenance drawn into an expression of great anxiety; gritting of teeth; faint, flickering pulse barely perceptible at the wrist. The blood seems to accumulate in the great cavities of the body, apparently driven there by contraction of the vaso-motor system—leaving the surface blanched and cold. During this cold stage of from thirty minutes' to several hours' duration, a thermometer introduced into the rectum would show a temperature of from one degree to several degrees above normal. After a time during which death seems to be imminent, the rigor gives away; a warm glow spreads over the body; the skin, from being blanched, takes on a rosy hue; a deep red flush suffuses the countenance; the heart reacts; and the patient, if conscious, complains of being uncomfortably hot, with great thirst, muscular pains and headaches. Often there occurs a slight discharge of urine of offensive odor and deep wine color. In a few hours a profuse perspiration begins; the fever subsides, temperature receding to subnormal, thirst abates; and all the uncomfortable symptoms which seem to have brought the patient so near the grave, cease. But for the weakness—a sort of languishing lassitude—he feels almost well. The contrast of his present comfortable condition with his appearance a few hours before is striking. At about the same hour, or a little earlier, next day, or maybe the day following the next, all the distressing symptoms reoccur with greater intensity. No one who has seen cases of congestive chill need be told of the great danger to life in first attack; the second attack, unless modified by treatment, is still more fatal; from the third, if permitted to occur, none recover—all die. From the beginning to the end of the attack, some patients remain in a profound coma—totally oblivious to all surroundings. The pupils of the eyes are largely dilated, irresponsive to light or, in exceptional instances, contracted; breathing is stetorous. In addition to profound coma, the head and feet may be drawn backward, causing the body to resemble a bow. This opisthotonous suggests meningitis. In the choleraic form, a person who has not been well for some time—especially morning and evening—is suddenly attacked with violent cramping pains in stomach and bowels; vomiting and diarrhea ensue without giving relief; consuming thirst possesses the patient. All liquids ingested are either promptly rejected through the mouth or rapidly pass off through the bowels. The bending, twisting, striving and writhing of the tortured patient is pitiful. The countenance soon assumes a weazened, starvation-like aspect; the eyes drawn back in the sockets; the lips thin and blue; the nose and chin peaked; the skin drawn tightly over the brow; the cheek bones prominent—exactly as I have seen in cases of true Asiatic cholera. The vomit may be a black, grumous material and the discharges from the bowels, when the solid contents have passed off, resemble rice water. Unless relief is prompt the patient dies. The physician who, for the first time, is confronted with this kind of case is apt to suspect that in some mysterious manner a corrosive poison has been ingested.

In another form of pernicious malarial fever, the skin and sclerotic membrane assume a distinctly orange color; or, sometimes, a dark, or brownish orange; the urine, a blood-like appearance. The serum drawn by blisters and the secretions are of a yellowish cast. This discoloration may be continuous; or it may continue for twenty-four or forty-eight hours and disappear, to reappear at stated periodical intervals. The patient may be fully or partially conscious; or, profound insensibility may exist from beginning to termination. The fever is apt to take on the same periodicity as the discoloration. This form is called hemoglobinuric fever by the profession, and "black jaundice" by the people. It often proves fatal. Sometimes it is confounded with yellow jaundice; obstruction of the gall duct; yellow fever; and some other maladies. The coloring matter is derived from decomposition of blood corpuscles.

It will be readily perceived that pernicious malarial fever, like hysteria, similates many other diseases. In plain typical cases occurring at the time and in the places expected, the diagnosis may present no difficulties; but there can be no question that many cases of pernicious malaria pass through the hands of the physician to the grave, unrecognized, under the name of congestion, typhoid, meningitis, cholera morbus, etc. Some of these cases, if promptly recognized and properly treated, would no doubt have recovered. Why is it that, in the same locality, at the same time, one physician sees a number of cases of remittent, continued fever, while his colleague treats an equal number of typhoid cases only. Where one finds meningitis, the other find the comatose form of malaria. This error is peculiarly unfortunate from this circumstance: If the people were plainly informed that their sons and daughters were suffering and dying from malarial infection they might be induced to adopt preventive measures.

Learned men, trained to accurate observation, state in popular works on practice that not infrequently the differential diagnosis of continued malarial fever from typhoid can not be made without the use of the microscope. My own observation, without a practical knowledge of microscopy, not only confirms the truth of this statement, but would extend it to other diseases similated by malaria—for instance, meningitis and cholera morbus. Hence, the importance of the microscope in diagnosis and treatment can hardly be over-estimated. Every physician should be skilled in its technique, or in convenient reach of a biological laboratory where blood examinations can be quickly made.

The surgeon who carelessly or ignorantly sets a broken limb at an abnormal angle may create a deformity which will constantly remind him of his error; but the physician who mistakes pernicious malarial fever for some other affection is likely to have his blunder concealed from mortal eyes by six feet of earth. The will of Providence or some other inscrutable reason may shield him from censure, and his own conscience, from the upbraidings of conscience.

It has often been observed that during and following an intensely malarious season, accidents and diseases are more frequent and more severe than in seasons of ordinary salubrity. Premature labors, abortions, menorrhagias, puerperal septicemia, mammary abscesses, pneumonias, bronchitis, etc., are more frequent and fatal; and chronic diseases do not so readily respond to the treatment usually found efficacious. It must not be forgotten that cases of multiple or double infections occur: as malaria and tuberculosis; malaria and pneumonia; malaria and cholera morbus; malaria and typhoid fever; and so on. In all cases of doubtful diagnosis it is safer to act on the presumption that somewhere lurks in the system the parasite, until the contrary has been proven.

In the *Journal of the American Medical Association,* August 27, 1904, there is condensed from the *Memphis Medical Monthly* a report of a case of mixed infection of typhoid and malaria. The diagnosis was established through microscopical examination of the blood.

In cases of multiple infection, the prognosis depends upon the complicating factors, the resisting powers of the patient and the intensity of the malarial infection. The condition of the vital organs at time of attack greatly influences the termination of the disease—for instance, a person laboring under valvular defects or artero-sclerosis may succumb to the first onset of a congestive malarial attack through heart failure or through apoplexy. The function of the defective organs may be sufficient for the ordinary purposes of life and yet be unable to withstand the shock of violent paroxysm.

In this way may be explained some sudden deaths occurring during the prevalence of malarial epidemics. In estimating the value of a course of treatment, due regard should be given to the condition of the patient during and immediately prior to the attack. To illustrate: A case of hemoglobinuric fever occurring in a patient with pre-existing renal insufficiency is almost sure to prove fatal under any treatment.

On visiting a case of pernicious malarial fever, the doctor is apt to find the bed of the patient surrounded by half a dozen or more frightened people earnestly engaged in rubbing with mustard, applying hot or cold applications in a very promiscuous way; while twenty-five or thirty more are excitedly looking on from the yard, the doors and cracks of the house; and at least one frantic female wildly shrieking for somebody to save her dear one. All eyes turn to the doctor. Expectation waits, tip-toe, for words of hope or despair. These circumstances are not conducive to the calm thinking and the deliberate action so much needed in times of great emergency.

The appalling nature of the symptoms suggests the strongest measures. Here let me recall a well-remembered remark of the much-lamented Prof. Austin Flint made many years ago to a class of medical students: "Never use a dangerous remedy when a harmless one will accomplish the purpose." With this precept well fixed in the mind, our first efforts will be directed to bring about reaction.

In the algid and in the choleraic forms, morphine admirably answers this purpose. The friction and applications may be allowed to go on in order to engage the attention of friends and to give the doctor time to think. A copious enema of pretty warm normal solution of salt may be administered high up through the colon tube; and a mild cathartic, not too drastic, given by the mouth, if practicable. But no time should be lost in getting into the patient's circulation a full dose of quinine—say fifteen grains for an adult—which should be repeated every four to six hours, until as much as one drachm has been used. The alimentary canal is usually in an unfavorable condition for absorption; medicine therefore, for immediate effect, if given by the mouth, might as well be thrown into the privy. The hypodermic method is urgently demanded. The objection to the use of quinine or whisky in this way is more than counterbalanced by the necessity for prompt action. Keeping in mind the etiological factor of the disease, there can be no excuse for failure to use the only reliable remedy for the destruction of the parasites. Let me illustrate by briefly quoting from Vol. III of *The Reference Handbook of Medical Sciences* a case reported by Dr. E. W. Schauffer, of Kansas City: "A woman sick for a week of malarial fever remained in a state of profound coma for seventy-two hours; it was impossible to arouse her in the least; pupils dilated and fixed; conjunctiva insensible to touch; breathing slow and stetorous; urine scanty and yellow; bowels not moved by enema and with what calomel could be forced through the mouth. She was as yellow as gold. This patient reacted and recovered under the persistent use, hypodermically, of quinine and whisky."

I have, myself, been more than once astonished at the recovery of patients from seemingly moribund conditions under this treatment. Physicians should always keep in their cases a solution of quinine for hypodermic use during the malarial season. I am aware that many cases of so-called "black jaundice," or hemoglobinuria, have recovered without the use of quinine. It may be in such cases that the malarial parasite has been jugulated by its own toxins or else destroyed by other excrementitious matter in the circulation. The problem must be left for solution to the new Texas doctor with his miscroscope. Of course, if there existed in the body of the patient no living parasites or toxins, it would be worse than useless to add the burdensome effects of quinine to an already over-burdened system. But in all cases of the yellow malarial disease where periodicity of symptoms indicate sporulation of the malarial parasite, quinine, above all others, is the remedy par excellence.

During the hot stage, cool affusions with ice bags to the head may reduce temperature and quiet nervous irritability. In some cases antipyretics may be cautiously used.

A physician once, over my protest, gave loose directions to the nurse to give a teaspoonful of a preparation containing aconite as long as the fever was high. A few hours later we were both hurriedly summoned to re-visit the patient—a well-made girl, that seemed to be getting on pretty well under an attack of simple remittent fever. Before arriving at the house, we were met by another messenger with the startling intelligence that the patient was dead. In this instance, the doctor failed to observe the Flint rule. He used aconite, a dangerous drug, carelessly, when a harmless remedy would have accomplished his purpose.

During convalescence, alimentation and tonics are to be relied on—iron and arsenic being especially valuable; but quinine must not be excluded from the treatment till full restoration of health. Whenever it is practicable, a change to a more salubrious climate should be advised.

There is much comfort in the thought that in the near future the new graduate, fresh from a four-years' course of careful training in the use of modern scientific methods of precision in diagnosis, will be able to clear up much of the obscurity which now veils these important morbid conditions.

